# Mobile clusters of single board computers: an option for providing resources to student projects and researchers

**DOI:** 10.1186/s40064-016-1981-3

**Published:** 2016-03-22

**Authors:** Christian Baun

**Affiliations:** Frankfurt University of Applied Sciences, Nibelungenplatz 1, 60318 Frankfurt am Main, Germany

**Keywords:** Single board computers, Performance, Speedup

## Abstract

Clusters usually consist of servers, workstations or personal computers as nodes. But especially for academic purposes like student projects or scientific projects, the cost for purchase and operation can be a challenge. Single board computers cannot compete with the performance or energy-efficiency of higher-value systems, but they are an option to build inexpensive cluster systems. Because of the compact design and modest energy consumption, it is possible to build clusters of single board computers in a way that they are mobile and can be easily transported by the users. This paper describes the construction of such a cluster, useful applications and the performance of the single nodes. Furthermore, the clusters’ performance and energy-efficiency is analyzed by executing the High Performance Linpack benchmark with a different number of nodes and different proportion of the systems total main memory utilized.

## Background

For student projects and research projects, dealing with parallel applications or private cloud services, an option for implementing clusters of inexpensive nodes would be useful. It may also be useful for small and medium-sized enterprises. Possible fields of application are among others the hosting of distributed database systems and clusters of network services like HTTP servers. Compared with commodity hardware servers, such clusters are an opportunity for reducing purchase costs for new hardware and the hardware related operating costs. In addition, such clusters can be constructed in a way that they can easily transported by the users because of their low weight and compact design. A scenario, where mobility is a beneficial characteristic, is when clusters shall be lend out to students. This way, they can solve exercises or do project work at home or some other place.

This paper is organized as follows. In “Options for resource provisioning” section, options for providing resources to student projects or research projects with limited financial resources are discussed.

Section “Related work” contains a discussion of related work and explains the reason for the construction of the clusters of mobile single board computers.

Section “Cluster of raspberry Pi nodes” presents a list of components of a mobile cluster of single board computers and a calculation of the energy costs.

Useful application scenarios for clusters of single board computers are analyzed in section “Useful applications”.

Section “Performance of the single board computers and the network infrastructure” contains an analysis of the performance of the CPU, storage and network interface of the single nodes.

In section “Analyzing the clusters’ performance with the HPL”, the performance and speedup of the entire cluster of single board computers is analyzed, by using the High Performance Linpack (HPL).

Section “Analysis of the energy-efficiency” contains an analysis of the energy-efficiency of the cluster.

Finally, section “Conclusions and future work” presents conclusions and directions for future work.

## Options for resource provisioning

Clusters usually consist of servers, workstations or personal computers as nodes. Since the mid-1990s, especially at universities and research institutions, cluster systems are assembled by using commodity hardware computers and Ethernet local area networks. Sterling et al. (1995) built such a cluster with the Linux operating system in 1994 and called it Beowulf cluster (Gropp et al. 2002), becoming a blueprint for numerous scientific projects afterwards.

The purchase cost for physical server resources can be challenging for student projects or scientific projects. Decommissioned hardware can be acquired for little money, but they require much space and the maintenance is labor intensive.

Another fact, which must be taken into account are costs, which arise from running physical computer resources. These include electricity cost.

If it is not mandatory to operate hardware resources in-house, outsourcing them or using services instead is an option. Dedicated server offerings and public cloud infrastructure service offerings can be used to provide compute resources to students or researchers.

### Obstacles against public cloud and dedicated server offerings

In some countries, it is not common that students have a credit card. This can be an obstacle for using public cloud infrastructure services for student projects.

In contrast to cloud infrastructure service offerings, which can be used on an on-demand-basis according to the pay-as-you-go principle, dedicated server offerings usually have a minimum rental period of at least a month. Depending on the number of systems, which are required to realize a specific distributed system, using dedicated server offerings may be an expensive choice.

A drawback of both dedicated servers and public cloud infrastructure services is the lack of a physical representation, which e.g. for students complicates understanding the functioning of distributed systems.

Building clusters of single board computers is another option for providing compute and storage resources to students and researchers for running and implementing distributed systems.

Table 1 contains a selection of single board computers, which provide sufficient computing power and main memory capacities to operate a Linux operating system and the required server daemons to implement clusters or private cloud services.

**Table 1:** Selection of single board computers

	BananaPi	ODROID-U3	Raspberry Pi B/B+	Raspberry Pi 2 B
CPU	ARM Cortex A7	ARM Cortex A9	ARM 11	ARM Cortex A7
CPU cores (#)	2	4	1	4
Clock rate	900 MHz	1700 MHz	700 MHz	900 MHz
RAM	1024 MB	2048 MB	512 MB	1024 MB
Ethernet interface	1000 Mbit	100 Mbit	100 Mbit	100 Mbit
Storage interfaces	SD, SATA	microSD, eMMC	SD/microSD	microSD

## Related work

Clusters of single board computers have already been implemented. Cox et al. (2009) assembled in the project Iridis-pi at the University of Southampton a cluster of 64 Raspberry Pi nodes with 256 MB main memory per node. The aim of this project was among others to implement an affordable cluster for running MPI^1^ applications and to evaluate the computational performance, network performance and storage performance of a cluster of single board computers.

Similar works have been done by Kiepert (2013), who assembled a Beowulf cluster by using 32 Raspberry Pi nodes with 512 MB main memory per node, at the Boise State University. He created a solid structure by using plexiglass, in which the cluster and its network and electric power infrastructure is housed. To power the single board computers, he used two standard PC power supplies and attached them by using one of the 5 V pins of the I/O header, each Raspberry Pi node provides. For the cooling of the cluster and mainly the power supplies, the cluster is equipped with 120 mm fans. The performance and speedup of this cluster was measured with an MPI-program that calculates *π* using the Monte Carlo method. Because this program can be parallelized very well, the speedup of the cluster is close to the theoretical maximum.

Abrahamsson et al. (2013) presented their work of building an affordable and energy-efficient cluster of 300 Raspberry Pi nodes, as well as several challenges and a number of opportunities.

Tso et al. (2013) presented a scale model of a Data Center, composed of clusters of 56 Raspberry Pi Model B nodes, that emulates the layers of a cloud stack (the focus is resource virtualisation to network management) by using Linux containers and the supporting LXC suite. The work compares the acquisition cost, electricity costs and cooling requirements of the cluster of single board computers with a testbed of 56 commodity hardware servers.

Various projects successfully built clusters of single board computers for implementing multi-node Hadoop installations mainly for educational purposes.

Jamie Whitehorn presented^2^ in 2013 a Hadoop cluster of five Raspberry Pi Model B nodes. For a productive usage, the performance of the Hadoop cluster is considered too slow. Especially the poor memory capacity is problematic for using Hadoop, but for educational purposes, regarding Hadoop itself, the system is well suited.

Also in 2013, the developers of the Cubieboard single board computer presented^3^ a Hadoop cluster of eight nodes. In contrast to the Raspberry Pi Model A and B, the Cubieboard computer used are equipped with a faster CPU (1 GHz clock rate) and a bigger main memory of 1 GB. Because of these resource characteristics, the Cubieboard were in 2013 better suited for deploying Hadoop.

Kaewkas and Srisuruk (2014) built at the Suranaree University of Technology a cluster of 22 Cubieboards, running Hadoop and Apache Spark, which are both open source frameworks for big data processing. The focus of their work is the I/O performance and the power consumption of the cluster.

Benefits of the single board computer cluster, used for this project, are the weight, which is only 7.8 kg, the reduced energy consumption (see Cluster of raspberry Pi nodes section) and that the entire cluster occupies little space and can easily be transported by the users, because all components are stored inside an aluminum hard case. This is an important feature because it allows to use the cluster for practical exercises close to the students and the cluster can easily be borrowed.

A further positive characteristic is that the cluster do not contain moving parts, such as fans or hard disk drives. The lack of moving parts makes the cluster less error prone.

## Cluster of Raspberry Pi nodes

**Fig. 1:**
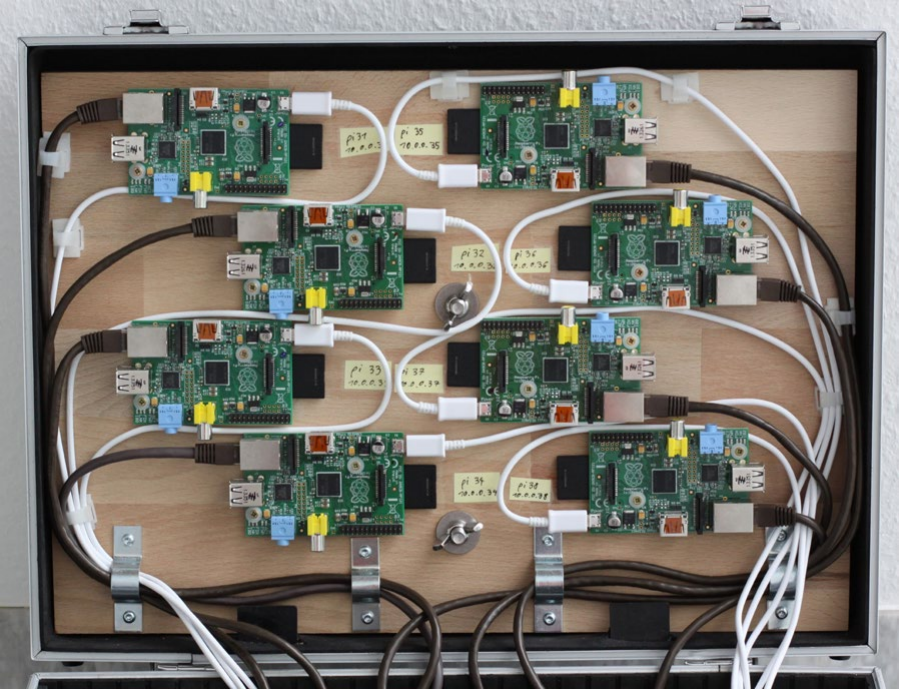
Eight Raspberry Pi Model B are the cluster nodes

**Fig. 2:**
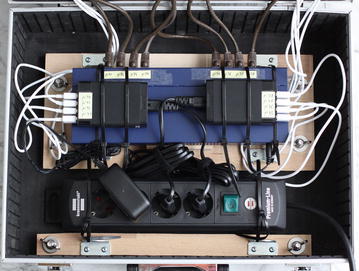
Power supply and network infrastructure of the cluster

In order to examine the performance and power consumption, a cluster (see Figs. 1, 2) of the following components was constructed:

- 8x Raspberry Pi Model B
- 8x SD flash memory card (16 GB each)
- 10/100 network switch with 16 ports
- 8x network cable CAT 5e U/UTP
- 2x USB power supply 40 W (5 V, 8 A)
- 8x USB 2.0 cable USB-A/Micro-USB
- Aluminum hard case 450x330x150 mm
- Power strip with at least 3 ports
- Various wooden boards, screws, cable ties, angles, wing nuts, etc.

The purchase cost for all components were approximately 500 €. The cluster is stored in an aluminum hard case to facilitate transporting and storing the system. A 100 Mbit Ethernet switch is sufficient for Raspberry Pi nodes with their 100 Mbit Ethernet interface.

Initially, the power supply was realized via individual USB power supplies (Samsung ETA-U90EWE, 5.0V, 2.0A) for each node, which were later replaced by two 5-port USB power supplies (Anker Model 71AN7105 40 W, 5.0 V, 8.0 A). Table 2 shows the power consumption of the cluster in idle operation mode and in stress mode^4^. Using just two power supplies causes lesser energy loss, which results in a reduced energy consumption.

**Table 2:** Power consumption of the cluster with eight Raspberry Pi Model B

	Idle mode	Stress mode
2x 5-port USB power supplies	$\approx$ 24 W	$\approx$ 26 W
8x 1-port USB power supplies	$\approx$ 31 W	$\approx$ 33 W

The energy costs per year ($C_Y$) for a 24/7 usage for a specific power consumption in kW during operation ($E$) can be calculated with Eq. (1). In the equation, energy costs of 0.25 € per kWh are assumed.

1 $$C_{Y} = {\text{E}} \times 24 \frac{{\text{hour}}}{{\text{day}}} \times 365.25 \frac{{\text{days}}}{{\text{year}}} \times \frac{0.25\,\,\EUR }{\text{ kWh }}$$

In a scenario where a cluster with eight Raspberry Pi Model B nodes and two 5-port USB power supplies runs all the time in stress mode (26 W energy consumption), the energy cost for 24/7 usage is approximately 56.98 € per year.

## Useful applications

A cluster of single board computers has very limited resources and cannot compete with the performance of higher-value systems. But despite these drawbacks, useful application scenarios exist, where clusters of single board computers are a promising option. This applies in particular for small and medium-sized enterprises as well as for academic purposes like student projects or research projects with limited financial resources.

### Private cloud infrastructure services

Different sorts of cloud services exist, which belong to the Infrastructure as a Service (IaaS) delivery model. One group of services allows the operation of virtual server instances and management of network resources. Popular public cloud IaaS offerings are among others the Amazon EC2, Google Compute Engine, GoGrid and Rackspace Cloud. Examples of private cloud IaaS solutions are OpenStack Nova, Eucalyptus (Nurmi et al. 2008), Nimbus (Keahey et al. 2009) and Apache CloudStack. These services have in common that they require a hypervisor like KVM (Kivity et al. 2007) or Xen (Barham et al. 2003). All evaluated single board computers (see Table 1) implement the ARM architecture and despite the fact, that numerous efforts like Dall and Nieh (2014) and Hwang et al. (2008) have been made to port KVM and Xen to this architecture, the computing power and main memory resources of the tested single board computers are not sufficient for server virtualization in a useful scale.

Further services, which belong to the IaaS family are object-based storage services like the public cloud offerings Simple Storage Service and Google Cloud Storage. Examples for private cloud solutions are Eucalyptus Walrus (Nurmi et al. 2009), Nimbus Cumulus (Bresnahan et al. 2011), OpenStack Swift and Riak S2. These service solutions can be executed on single board computers. In a cluster of single board computes, each request to a object-based storage service creates little workload on a node. Eucalyptus Walrus, OpenStack Swift and Riak S2 even implement replication over multiple nodes.

### Private cloud platform services

A Platform as a Service (PaaS) implements a scalable application runtime environment for programming languages. The target audience are software developers and end users who like to provide and consume services in a corresponding market place. A PaaS allows to scale from a single service instance to many, depending on the actual demand (Armbrust et al. 2009). Prominent instances of public cloud PaaS offerings are Google App Engine, Microsoft Azure Platform and Engine Yard.

In some cases, it might be desirable to avoid public cloud offerings for privacy or legal reasons for example. Advantageously, private cloud solutions exist. Examples are AppScale (Chohan et al. 2009; Bunch et al. 2010), Apache Stratos and OpenShift. Running these services is potentially possible in a cluster of single board computers as long as no virtualization layer (hypervisor) is required.

### Distributed file systems

Two different types of distributed file systems exist:

1. Shared storage file systems, which are also called shared disk file systems
2. Distributed file systems with distributed memory

Clusters of single board computers are an inexpensive option for testing and developing distributed file systems with distributed memory. Examples for such file systems are Ceph (Weil et al. 2006) GlusterFS^5^, HDFS (Borthakur 2008), PVFS2/OrangeFS (Carns et al. 2000) and XtreemFS (Hupfeld et al. 2008).

In order to use shared storage file systems like OCFS2 (Fasheh 2006) and GFS2, all nodes must have direct access to the storage via a storage area network (SAN), e.g. implemented via Fibre Channel or iSCSI. Connecting the nodes of a cluster of single board computers with a SAN is an option with two major drawbacks. First, the purchase cost of a SAN infrastructure would in most cases be higher as the sum of all other cluster components (including the nodes itself). Second, the Fibre Channel interface cards, often called host bus adapters (HBA), are usually connected via PCI Express or Thunderbolt. None of these interfaces are provided by any of the evaluated single board computers. Using iSCSI via Ethernet is not a recommendable option for single board computers (see Table 1) which provide just a single Ethernet interface with a maximum data rate of 100 Mbit/s.

### Distributed database systems

Numerous free database systems support cluster operation to provide a higher level of availability and a better performance for query and data modification operations compared with single node operation. Examples of distributed database systems, which have been successfully tested on clusters of single board computers are the document-oriented databases Apache CouchDB^6^, the column-oriented database Apache Cassandra^7^, the key/value database Riak^8^, as well as the relational database management system MySQL^9^.

Further relational database management systems and NoSQL database systems support cluster operation mode and should be deployable in a cluster of single board computers in principle.

### High-throughput-clustering

Single board computers provide sufficient resources for running network services like HTTP servers, mail servers or FTP servers. Each request to such a service creates little load on a node.

To realize e.g. a High-Throughput-Cluster of HTTP servers, only a server software and the load balancer functionality are required. As HTTP server software, the Apache HTTP Server or a resource-saving alternative like Nginx or Lighttpd can be deployed. The Apache server software provides the load balancer module mod_proxy_balancer^10^ and the Nginx server software implements load balancing functionality too. Another option is using a load balancing solution like Ultra Monkey, which can be operated in a redundant way by running a stand-by instance.

Detailed monitoring of the state and load of the single nodes can be implemented with monitoring tools like Nagios and Ganglia.

### High-performance-clustering and parallel data processing

Clusters of nodes with physical hardware are the ideal environment to test and develop parallel applications for High-Performance-Clusters, because no virtualization layer and additional guest operating systems influence the performance of the nodes.

For the distributed storage and parallel processing of data, the Apache Hadoop framework, which implements the MapReduce (Dean and Ghemawat 2004) programming model, can be used.

Solutions for implementing parallel applications are among others MPI, PVM^11^, OpenSHMEM and Unified Parallel C (Dinan et al. 2010), which all can be used in clusters of single board computers.

## Performance of the single board computers and the network infrastructure

The performance of the CPU, the local storage and the clusters’ network infrastructure was measured in order to get an impression about the performance capability of a single node of the cluster.

### CPU performance

The benchmark suite SysBench was used to measure the CPU performance. Table 3 shows the total execution time of the benchmark, while testing each number up to value 10,000 if it is a prime number.

For comparison, not only the CPU performance of the Raspberry Pi Model B, used in the cluster was measured, but also of the BananaPi and the ODROID-U3. Furthermore, the benchmark was executed on a Lenovo ThinkPad X240 notebook with an Intel i7-4600U quad-core CPU.

The benchmark scales well on multiple nodes. The measurement results in Table 3 show that doubling the number of cores nearly halves the execution time.

Increasing the clock rate of the Raspberry Pi from 700 to 800 MHz does not require overvolting the CPU and results in a noticeable increase of the processing power. For this reason, the Raspberry Pi nodes of the cluster are overclocked to 800 MHz.

The measurement results in Table 3 also show that for the BananaPi and the ODROID-U3, using more threads than CPU cores available does not result in a significant performance gain. For the Raspberry Pi, the execution time is even extended, because of the additional overhead, that results of the increased number of context switches.

**Table 3:** Total execution time of SysBench benchmark, testing each number up to value 10,000 if it is a prime number

	CPU cores (#)	Total execution time with...					
		Clock rate	1 thread	2 threads	4 threads	8 threads	16 threads
Raspberry Pi Model B	1	700 MHz	503.01 s	503.98 s	504.19 s	504.30 s	504.45 s
Raspberry Pi Model B	1	800 MHz$^{*}$	439.68 s	440.47 s	440.56 s	440.75 s	440.82 s
BananaPi	2	900 MHz	292.98 s	147.74 s	147.85 s	147.82 s	147.82 s
ODROID-U3	4	1.7 GHz	133.88 s	66.90 s	34.28 s	33.93 s	33.91 s
ThinkPad X240	4	2.1 GHz	9.72 s	5.35 s	3.03 s	2.95 s	2.97 s

$^{*}$ Overclocking the Raspberry Pi Model B to 800 MHz is possible without overvolting the CPU

### Storage performance

Only a limited number of options exist to attach local storage to the cluster nodes. The Raspberry Pi provides (depending on the model) two or four USB interfaces, connected via an internal USB 2.0 hub and an interface for secure digital cards (SD cards). By using an appropriate passive adapter, microSD flash cards can be used as well. Each Raspberry Pi node in the cluster is equipped with a 16 GB flash storage card, which stores the operating system and provides storage capacity for applications and data.

#### Sequential read/write performance

The sequential read/write performance of the local storage of a single cluster node was measured with the tool dd, while reading and writing a single file of size 300 MB. Several (micro)SD storage cards of different manufacturers and speed classes^12^ were tested. To avoid interferences, caused by the page cache, it was dropped before each read performance test and the flag oflag=sync was used for write performance tests with the dd command. The file system used was ext4 with 4 kB block size and journaling mode ordered, which implies that all file data are directly written into the file system prior to its metadata being committed to the journal. The values in Table 4 are averages of five measurement rounds.

**Table 4:** Sequential read/write performance on local storage on a single Raspberry Pi Model B node and on a ThinkPad X240

Manufacturer	Form factor	Capacity [GB]	Speed class	Product ID/Item number	Read$^{1}$ performance [MB/s]		Write$^{2}$ perf. [MB/s]	
					Raspberry Pi Model B	ThinkPad X240	Raspberry Pi Model B	ThinkPad X240
Kingston	SD	16	4	SD4/16GB	17.50	20.58	4.36	4.38
SanDisk	SD	16	4	SDSDB-016G	15.80	22.40	4.36	4.46
Verbatim	SD	16	4	#44020	18.40	22.18	7.48	7.62
Verbatim	microSD	16	4	#44007	18.60	71.92	9.50	10.86
Samsung	microSD	16	6	MB-MS16D	18.40	21.92	10.30	12.06
Kingston	SD	16	10	SD10V/16GB	17.50	34.34	10.60	9.82
Kingston	microSD	16	10	SDC10/16GB	17.72	33.64	8.38	11.60
Samsung	microSD	16	10	MB-MP16D	18.10	40.86	10.12	12.24
SanDisk	SD	16	10	SDSDL-016G	18.68	42.04	11.52	12.78
SanDisk	microSD	16	10	SDSDQUI-016G	18.70	42.72	12.18	13.26
SONY	SD	16	10	SF-16UX	17.30	59.10	14.28	24.00
SONY	microSD	16	10	SR-16UY	17.90	35.16	9.64	10.98

$^{1}$ Measured with: echo 3 ${\mathtt{>}}$ /proc/sys/vm/drop_caches&& dd if=/tmp/testfile of=/dev/null bs=300M count=1

$^{2}$ Measured with: dd if=/dev/zero of=/tmp/testfile bs=300M count=1 oflag=sync

When used with the Raspberry Pi, most tested class 6 and class 10 flash storage drives provide a significant better data rate for sequential write compared with the class 4 drives. The sequential read performance of all tested drives is limited by the maximum data rate of the storage card interface, the Raspberry Pi Model B is equipped with. The SD card interface on the Raspberry Pi implements a 4-bit data bus and 50 MHz clock rate. Therefore, the theoretical maximum data rate is 25 MB/s, which cannot be reached in practice. For comparison, the data rate of the flash storage drives for sequential read and write was also measured with the internal Realtek RTS5227 PCI Express card reader of a Lenovo ThinkPad X240 notebook. The results in Table 4 show that the maximum data rate of the tested class 10 drives for sequential read is more than double the data rate, the drives provide in the Raspberry Pi.

Measuring the sequential read and write performance is a procedure, which is quite common for benchmark purposes, but its significance for practical applications is limited because reading and writing large amounts of data in row is carried out quite seldom on many systems. Use cases are e.g. streaming media and the up- and download of objects, which are at least several MB in size. More common in root file systems and during operation of e.g. HTTP servers and database systems are random read and write operations.

#### Random read/write performance

**Table 5:** Random read performance on local storage on a single Raspberry Pi Model B node

Manufacturer	Form factor	Capacity [GB]	Speed class	Random read performance$^{1}$ [kB/s] with record size [kB]...								
				4	8	16	32	64	128	256	512	1024
Kingston	SD	16	4	3892	5103	7989	10,494	14,117	15,491	17,275	18,040	18,110
SanDisk	SD	16	4	3367	5567	8231	11,492	14,593	16,302	17,622	18,257	18,516
Verbatim	SD	16	4	4542	6943	10,561	13,329	15,501	17,072	18,016	18,216	18,551
Verbatim	microSD	16	4	4665	7308	11,368	14,198	16,041	17,524	18,262	18,657	18,674
Samsung	microSD	16	6	3910	6691	9654	13,009	15,938	16,926	18,003	18,520	18,570
Kingston	SD	16	10	3822	5790	8899	11,699	14,622	15,895	17,517	18,130	18,171
Kingston	microSD	16	10	3905	5416	8644	12,018	14,530	16,594	17,521	18,214	18,279
Samsung	microSD	16	10	3973	6754	10,264	13,377	15,522	17,193	18,073	18,562	18,586
SanDisk	SD	16	10	4140	6420	9553	12,043	14,835	16,942	17,647	18,395	18,562
SanDisk	microSD	16	10	3991	6406	9564	12,553	15,195	16,884	17,899	18,428	18,616
SONY	SD	16	10	5061	5667	8503	11,164	13,900	15,996	17,342	18,046	18,127
SONY	microSD	16	10	4026	6079	9169	11,951	14,802	16,429	17,619	18,186	18,255

$^{1}$ Measured with: iozone -RaeI -i 0 -i 1 -i 2 -y 4k -q 1M -s 500m -o -f /tmp/testfile

For measuring the random read and write performance, the benchmark tool iozone v3.430 was used. The results show that all tested SD cards provide even for record size 4 kB a random read performance of 3-5 MB/s (see Table 5). Increasing the record size increases the data rate until the performance of sequential read is reached.

The performance for random write (see Table 6) is significantly lower compared with sequential write. The random write performance of SD cards is caused by the internal architecture of this type of flash storage. Memory cells of NAND flash storage are grouped to pages and so called erase blocks. Typical page sizes are 4, 8 or 16 kB. Although it is possible for the controller to write single pages, the data cannot be overwritten without being erased first and an erase block is the smallest unit that a NAND flash storage can erase. The size of the erase blocks of SD cards is typically between 64 or 128 kB. In modern SD cards, small numbers of erase blocks are combined into larger units of equal size which are called allocation groups or allocation units or segments. The usual segment size is 4 MB. The controllers of SD cards implement a translation layer. For any I/O operation, a translation from virtual to physical address is carried out by the controller. If data inside a segment shall be overwritten, the translation layer remaps the virtual address of the segment to another erased physical address. The old physical segment is marked dirty and queued for an erase. Later, when it is erased, it can be reused. The controllers of SD cards usually cache a single or more segments for increasing the performance of random write operations. If a SD card stores a root file system, it is beneficial if the controller of the card can cache the segment(s) where the write operation(s) takes place, the segments, which store the metadata for the file system and (if available) the journal of the file system. Consequently, the random write performance of a SD card depends among others of the erase block size, the segment size and the number of segments, the controller caches.

A significant performance advantage of the tested class 10 flash storage cards compared with the tested class 4 or class 6 flash storage cards is not visible.

**Table 6:** Random write performance on local storage on a single Raspberry Pi Model B node

Manufacturer	Form factor	Capacity [GB]	Speed class	Random write performance$^{1}$ [kB/s] with record size [kB]...								
				4	8	16	32	64	128	256	512	1024
Kingston	SD	16	4	81	6	13	26	52	103	207	415	834
SanDisk	SD	16	4	104	85	17	35	70	140	280	536	1076
Verbatim	SD	16	4	77	161	335	594	902	875	1335	1832	2977
Verbatim	microSD	16	4	121	257	586	962	1272	1780	2310	2894	3417
Samsung	microSD	16	6	133	272	631	1055	1485	2028	2592	2994	3473
Kingston	SD	16	10	200	10	21	43	86	176	355	737	1551
Kingston	microSD	16	10	224	13	26	53	108	219	446	917	1939
Samsung	microSD	16	10	128	263	581	1006	1440	2018	2589	3043	3430
SanDisk	SD	16	10	323	451	40	81	162	329	665	1327	2749
SanDisk	microSD	16	10	334	419	41	83	167	336	672	1345	2778
SONY	SD	16	10	208	11	23	47	94	189	377	751	1469
SONY	microSD	16	10	216	12	25	51	102	207	420	845	1807

$^{1}$ Measured with: iozone -RaeI -i 0 -i 1 -i 2 -y 4k -q 1M -s 500m -o -f /tmp/testfile

#### Further options for local storage

The available USB interfaces provide further options to implement a local storage. It is possible to attach a single or multiple hard disk drives (HDD) or flash storage drives via USB. Patterson et al. (1988) described that if multiple drives are attached, the performance and availability can be improved by combining them to redundant arrays of independent disks (RAID).

No matter what storage technology is used, the USB 2.0 interface limits the possible throughput. Solid state drives (SSD) and HDDs provide enough performance for read and write to utilize the entire transfer capacity of the USB 2.0 interface. Drawbacks of attaching SSDs or HDDs on each cluster node are higher purchase cost for the cluster and increased energy consumption.

Mordvinova et al. (2009) showed that RAID arrays of USB flash storage drives can be purchased for less cost, compared with SSDs or HDDs, but like SD cards they usually provide a poor performance for random write operations. Therefore, USB flash storage drives are a useful option mainly for read-mostly applications like storing the content of web servers and for CPU bound applications.

For these reasons, only SD cards are used in the cluster of single board computers.

### Network performance

The network performance between the nodes was measured with the command-line tool iperf v2.0.5 and with the NetPIPE v3.7.2 benchmark.

According to iperf, the network performance between the nodes of the cluster is 76-77 Mbit per second.

**Fig. 3:**
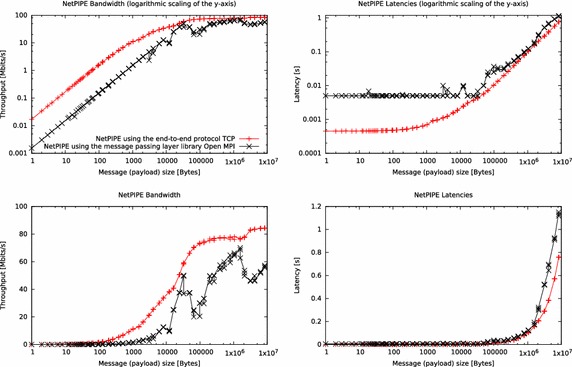
Analysis of the network performance inside the cluster by using the NetPIPE benchmark

A more detailed analysis of the network performance is possible with the NetPIPE benchmark. It tests the latency and throughput over a range of message sizes between two processes. The benchmark was executed inside the cluster one time by just using TCP as end-to-end (transport layer) protocol and one time by using the Open MPI message passing layer library. The results in Fig. 3 show that the smaller a message is, the more is the transfer time dominated by the communication layer overhead. For larger messages, the communication rate becomes bandwidth limited by a component in the communication subsystem. Examples are the data rate of the network link, utilization of the transmission medium or a specific device between sender and receiver like the network switch inside the mobile cluster.

As described by Snell et al. (1996) and clearly evident in Fig. 3, using MPI (which also uses TCP as transport layer protocol) is an overhead that has a negative impact on throughput and latency. The best measured throughput when using MPI is 65 Mbit per second. When using just TCP, the throughput reaches up to 85 Mbit per second.

As long as the payload fits into a single TCP segment, the latency when using MPI is approximately ten times worse compared when using just TCP. The maximum transmission unit (MTU), which specifies the maximum payload inside an Ethernet frame, is 1500 Bytes in our cluster. Consequently, the maximum segment size (MSS), which specifies the maximum payload inside a TCP segment, is 1460 Bytes.

The drop of the data rate when using MPI at around 64 kB payload size is caused by the MPI library that implements the asynchronous eager protocol and the synchronous rendezvous protocol. While eager does not await an acknowledgement prior starting a send operation, the rendezvous does. This is because of the assumption that the receiver process can store small messages in its receive buffer any time. The default size limit, where the installed Open MPI library sends messages via eager protocol is 64 kB.

Further drops of the data rate when using MPI, especially around 4 MB payload size are probably caused by the limited CPU resources of the Raspberry Pi nodes. When executing the MPI benchmark with such a message size, the CPUs of the nodes are almost entirely utilized.

**Fig. 4:**
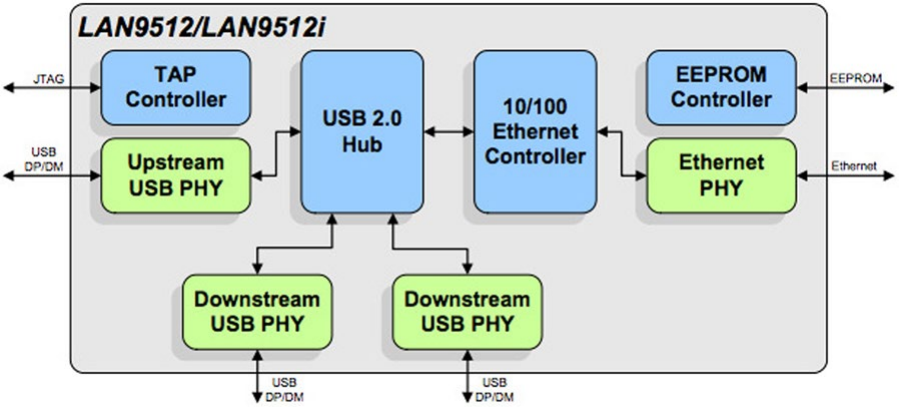
The Ethernet controller of the Raspberry Pi is connected with the USB 2.0 hub. Image source: SMSC (see footnote 13)

The poor overall Ethernet performance of the Raspberry Pi nodes is probably caused by the fact, that the 10/100 controller is a component of the LAN9512 controller^13^. This chip contains the USB 2.0 hub and a built-in 10/100 Mbit Ethernet controller, which is internally connected (see Fig. 4) with the USB hub.

## Analyzing the clusters’ performance with the HPL

Besides analyzing the performance of individual nodes and their resources, it is interesting to examine the performance of the cluster as a whole.

The High Performance Linpack (HPL)^14^ benchmark is an established approach to investigate the performance of cluster systems. It is among others used by the Top500 project, which maintains a list of the 500 most powerful computer systems. As described by Luszczek et al. (2005) and Dunlop et al. (2010), the benchmark solves a linear system of equations of order $n$

$$\begin{aligned} A \times x = b; \qquad A \in {\mathbb {R}}^{n\times n}; \quad x,b \in {\mathbb {R}}^{n} \end{aligned}$$

**Fig. 5:**
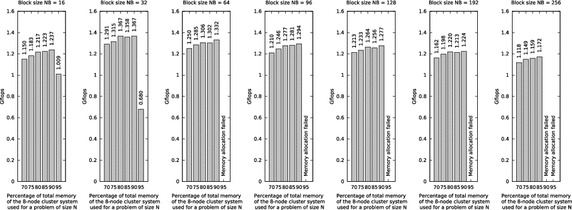
Analysis of the clusters Gflops performance, when using all eight nodes, by using the HPL benchmark with different values for the parameter $NB$ and different proportions of the systems total main memory utilized. The concrete values for problem size $N$ can be seen in Table 7

that is divided into blocks of size $P \times Q$, by using double-precision (8 Bytes) floating-point arithmetic (Gaussian elimination with partial pivoting) on computer systems with distributed memory. The execution of the HPL can be specified manually in the config file HPL.dat with several parameters. Some tools like the Top500 HPL Calculator^15^ are helpful to find some initial settings, but finding the most appropriate settings for a specific system is not a simple task and takes some time.

$P \times Q$ is equal to the number of processor cores used. The developers of the HPL recommend^16^ that $P$ (the number of process rows) and $Q$ (the number of process columns) should be approximately equal, with $Q$ slightly larger than $P$. Consequently, in the cluster of eight Raspberry Pi single board computers, the values $P\,=\,1, Q\,=\,1$ were used for benchmarking just a single node, $P\,=\,1, Q\,=\,2$ for two nodes, $P\,=\,1, Q\,=\,4$ for four nodes and $P\,=\,2, Q\,=\,4$ for the entire cluster with eight nodes.

The parameter $$N$$ specifies the problem size—the order of the coefficient matrix. It is challenging to find the largest problem size that fits into the main memory of the specific system. Therefore, the main memory capacity for storing double precision (8 Bytes) numbers need to be calculated. Utilizing the entire main memory capacity for the benchmark is impossible because the operating system and the running processes still consume memory and using the swap memory has a negative impact on the performance. Thus, it is promising^17^$$^,$$^18^ to set $$N$$ to a value 80–90 % of the available main memory.

The problem size $N$ can be calculated with Eq. (2). It depends on the number of nodes in the system, the reduction coefficient $R$ which specifies how much percent of the entire main memory of the cluster shall be utilized by the benchmark and the main memory capacity $M$ of a single node in GB. The Raspberry Pi cluster nodes used for this project are equipped with 512 MB main memory. A part of the main memory is assigned as video memory to the GPU, which lacks own dedicated memory. Because in the cluster, the GPUs are not used at all, the minimal GPU memory was set, which is 16 MB. This results in 496 MB main memory left for the operating system and the applications on each node. After the operating system Raspbian and the daemon and client for the distributed file system is started, approximately 400–430 MB main memory remains available on each node.

2 $$\begin{aligned} N = \sqrt{\left( \frac{M * 1024 * 1024 * 1024 * P * Q}{8}\right) } * R \end{aligned}$$

If for example the value of $N$ shall be big enough to fill around 80 % of the memory capacity of four nodes ($P\,=\,1, Q\,=\,4$) of the cluster system, the calculation is as follows:

$$\begin{aligned} N= \sqrt{\left(\frac{0.496\,{\text{ GB }} * 1024 * 1024 * 1024 * 1 * 4}{8}\right)} * 0.8 \\\approx 13,054 \end{aligned}$$

A further important parameter is the block size $NB$. As optimization, $N$ should be $NB$ aligned$^{18}$. For this example, if we consider $NB\,=\,32$, we calculate $\frac{13,054}{32} \,=\, 407.9375 \approx 407$ and next $407 \times 32\,=\, 13,024\,=\,N$. For this work, the HPL benchmark was executed with different parameters in the cluster of single board computers. Figure 5 shows the Gflops when executing the benchmark with different values for the parameter $NB$ in the cluster system when using all eight nodes and utilizing different proportions of the systems’ total main memory. These tests were carried out to find a recommendable value for $NB$.

For $NB\,=\,16$ and $NB\,=\,32$, a performance drop is observed, when utilizing 95 % of the systems’ main memory. This is caused by the heavy use of swap memory.

The results in Fig. 5 show that from the tested values, $NB\,=\,32$ causes the best performance. For this reason, further performance investigations with the HPL benchmark were carried out with this value for the parameter $NB$.

### Analysis of the speedup

Table 7 shows the values of the parameters $N$, $P$, $Q$ and $NB$, as well as the runtimes, required to solve the linear system and the resulting Gflops^19^.

The benchmark was executed in the cluster with just a single node, two nodes, four nodes and eight nodes to investigate the speedup. The speedup $S_{P}$, that can be achieved when running a program on $P$ processors is defined as

3 $$\begin{aligned} S_{P} = \frac{F_{P}}{F_{1}} \end{aligned}$$

where $F_{1}$ is the Gflops on a single-processor system and $F_{P}$ is the Gflops on a multiprocessor system.

The theoretical maximum speedup is equal to the number of single-processor nodes, which means it is value 2 for two nodes, value 4 for four nodes, value 8 for eight nodes, etc.

The results in Table 7 show, that increasing the number of nodes also increases the speedup significantly. The best benchmark results were obtained, when $N$ is set to a value 80–90 % of the available main memory.

**Fig. 6:**
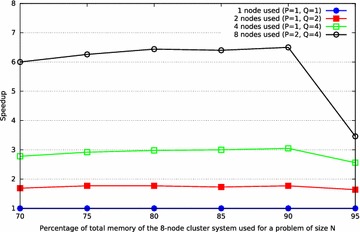
Analysis of the clusters speedup by using the HPL benchmark with $NB\,=\,32$, different numbers of nodes and different proportion of the systems total main memory utilized. The concrete values for problem size $N$ can be seen in Table 7

The low speedup, when utilizing 95 % of the systems’ main memory, is caused by the heavy use of swap memory. Figure 6 highlights this observation.

**Table 7:** Analysis of the clusters Gflops performance and speedup by using the HPL benchmark with $NB\,=\,32$, different numbers of nodes and different proportion of the systems total main memory utilized

Proportion of total main memory of the system utilized	N	Nodes used	NB	P	Q	Time to solve the linear system	Gflops$^{1}$	Speedup$^{2}$
$\approx$ 70 %	5696	1	32	1	1	589.17 s	0.209	1.00
	8064	2	32	1	2	983.76 s	0.355	$\approx$ 1.69
	11,392	4	32	1	4	1641.22 s	0.600	$\approx$ 2.78
	16,128	8	32	2	4	2166.95 s	1.291	$\approx$ 6,17
$\approx$ 75 %	6112	1	32	1	1	722.29 s	0.210	1.00
	8640	2	32	1	2	1153.95 s	0.372	$\approx$ 1.77
	12,224	4	32	1	4	1977.85 s	0.615	$\approx$ 2.92
	17,280	8	32	2	4	2617.08 s	1.315	$\approx$ 6.26
$\approx$ 80 %	6496	1	32	1	1	859.81 s	0.212	1.00
	9216	2	32	1	2	1503.04 s	0.347	$\approx$ 1.77
	13,024	4	32	1	4	2328.03 s	0.632	$\approx$ 2.98
	18,432	8	32	2	4	3055.37 s	1.367	$\approx$ 6.44
$\approx$ 85 %	6912	1	32	1	1	1037.84 s	0.212	1.00
	9792	2	32	1	2	1705.12 s	0.367	$\approx$ 1.73
	13,856	4	32	1	4	2782.23 s	0.637	$\approx$ 3.00
	19,584	8	32	2	4	3688.51 s	1.358	$\approx$ 6.40
$\approx$ 90 %	7328	1	32	1	1	1246.73 s	0.210	1.00
	10,368	2	32	1	2	1993.88 s	0.372	$\approx$ 1.77
	14,656	4	32	1	4	3274.85 s	0.641	$\approx$ 3.05
	20,768	8	32	2	4	4370.31 s	1.367	$\approx$ 6.50
$\approx$ 95 %	7744	1	32	1	1	1578.72 s	0.196	1.00
	10,944	2	32	1	2	2699.97 s	0.323	$\approx$ 1.64
	15,488	4	32	1	4	4927.79 s	0.502	$\approx$ 2.56
	21,920	8	32	2	4	10,326.42 s	0.680	$\approx$ 3.46

$^{1}$ The Gflops are rounded to three decimal places behind the decimal point

$^{2}$ The Speedup is rounded to two decimal places behind the decimal point

### Analysis of the efficiency

Especially for the previous mentioned Top500 list, two performance indicators are considered important. These are:

- $Rpeak$, which is the theoretical peak performance of the system. It is determined by counting the number of floating-point additions and multiplications (in double precision), that can be completed during a period of time, usually the cycle time of the machine (see footnote 17). The ARM 11, which is used by the Raspberry Pi computers, can process a floating-point addition in one cycle and requires two cycles for a floating-point multiplication^20^. The calculation of $Rpeak$ of a system is as follows:
  $$\begin{aligned} Rpeak\,{\text{[Gflops] }}= & {} {\text{ Clock }} {\text{ speed }} {\text{ per }} {\text{ core }} {\text{[GHz] }} \\&\times {\text{ Number }} {\text{ of }} {\text{ cores }} \\&\times {\text{ Operations }} {\text{ per }} {\text{ cycle }} \\ \end{aligned}$$
  Thus, the $Rpeak$ of a cluster of eight Raspberry Pi nodes (with 800 MHz clock speed) is 6,4 Gflops for floating-point addition operations and 3,2 Gflops for floating-point multiplication operations.
- $Rmax$, is the maximal performance that was achieved with the HPL. In case of our cluster, $Rmax$ has value 1.367 Gflops (see Fig. 5; Table 7).

The efficiency of a specific system in percent is calculated via $\frac{Rmax}{Rpeak}*100$. In case of our cluster, the efficiency depends of the executed operations and is only between $\approx 21$ % and $\approx 42$ %. The exact reason for this low efficiency was not evaluated. But as described by Luszczek et al. (2005), the HPC Challenge benchmark test suite stresses not only the processors, but the memory system and the interconnect too. Therefore, it is likely that the low network performance (see Network performance section), as well as the memory performance of the Raspberry Pi computers have a negative impact here.

## Analysis of the energy-efficiency

Knowing the clusters’ electric energy consumption (see Table 2) and its performance when executing the HPL benchmark (see Analyzing the clusters’ performance with the HPL section) is the precondition to analyze the clusters’ energy-efficiency.

The Green500 list, which is a complement to the Top500 list, uses the flops per Watt metric (Sharma et al. 2006) to rank the energy efficiency of supercomputers^21^. The metric is defined as

4 $$\begin{aligned} {\text{ flops }} {\text{ per }} {\text{ Watt }} = \frac{{Rmax\,{\text{[flops] }}}}{P(Rmax)\,{\text{[Watt] }}} \end{aligned}$$

$P(Rmax)$ is the average system power consumption while executing the HPL with a problem size that delivers $Rmax$. When executing the HPL benchmark, the power consumption of the cluster depends of the number of nodes used for the benchmark. The average system power consumption while executing the HPL is approximately 26 W when using all eight nodes. With $Rmax$ = 1.367 Gflops, the cluster provides approximately $52.57$ Mflops per Watt.

## Conclusions and future work

The performance of single board computers cannot compete with higher-value systems because the performance of their components, especially CPU, main memory and network interface. The same applies for clusters of single board computers. The maximum observed performance $Rmax$ of the cluster system, implemented for this work is 1.367 Gflops. This performance would be sufficient for 216th place in the Top500 list from June 1993. But compared with recent cluster sites this performance is very low. In the most recent Top500 list from June 2015, the last entry (500th place) provides $Rmax$ = 164,800 Gflops, which is more than factor $120,000$ better compared with our cluster.

Also the energy-efficiency cannot compete with higher-value systems. The cluster provides approximately $52.57$ Mflops per Watt, which would be sufficient for 186th place in the Green500 list from November 2007. In the most recent list from November 2015, the best entry (1st place) provides $7,031.58$ Mflops per Watt, which is more than factor $133$ better compared with our cluster.

Regardless of the performance or energy-efficiency, clusters of single board computers like the Raspberry Pi are useful for academic purposes and research projects because of the lesser purchase costs and operating costs compared with commodity hardware server resources. They can also provide the same or a better level of reliability compared with single server systems.

Since February 2015, the Raspberry Pi 2 is available for purchase. This single board computer provides more computational power and main memory compared with the cluster nodes, that were used in this project. Building a cluster of this computers is one of the next steps. It is interesting to discover how increasing the processor cores by factor four and doubling the main memory per node affects the performance because the available main memory per processor core is halved. It is further interesting to investigate if the increased CPU performance has a positive impact on the network performance when using MPI.

Further next steps are the implementation of clusters of different single board computers and comparing their performance.

## Footnotes

1 Message Passing Interface (MPI) is the de facto standard for distributed scientific computing. The MPI standard defines the syntax and semantics of the MPI functions. Several implementations of the standard exist. One example is MPICH.

2 The slides, presented by Jamie Whitehorn at the Strata + Hadoop world conference 2013 are accessible via the URL http://www.idatasci.com/uploads/1/4/6/6/14661274/jamiewhitehorn_raspberryflavouredhadoop_annotated.pdf.

3 Further information about the Hadoop cluster of Cubieboard computers provides the web page http://cubieboard.org/2013/08/01/hadoophigh- availability-distributed-object-oriented- platform-on-cubieboard/.

4 The nodes were put into stress mode by using the command-line tool stress. Further information about this tool provides the web page http://people.seas.harvard.edu/~apw/stress/.

5 Further information about GlusterFS provides the document *Cloud Storage for the Modern Data Center: An Introduction to Gluster Architecture* (2011), which is accessible via the URL http://moo.nac.uci.edu/~hjm/fs/An_Introduction_To_Gluster_ArchitectureV7_110708.pdf.

6 Further information about deploying CouchDB on a cluster of 12 Raspberry Pi computers provides the web page http://cloudant.com/blog/building-a-cloudant-cluster-of-raspberry-pis/.

7 Further information about deploying Apache Cassandra on a cluster of 32 Raspberry Pi computers provides the web page http://www.datastax.com/dev/blog/32-node-raspberry-pi-cassandra-cluster.

8 Further information about deploying Riak on a cluster of three Raspberry Pi computers provides the web page http://basho.com/posts/technical/building-a-riak-cluster-on-raspberry-pi/.

9 Further information about deploying MySQL on a cluster of Raspberry Pi computers provides the web page http://www.bigair.net/rpi.

10 Further information about the load balancing module mod_proxy_balancer provides the documentation for the Apache HTTP Server Version 2.2, which can be accessed via the URL http://httpd.apache.org/docs/2.2/mod/mod_proxy_balancer.html.

11 Parallel Virtual Machine (PVM) is a software which allows to connect heterogeneous computer systems to a single distributed parallel computer. Since the late 1990s, PVM is more and more superseded by MPI.

12 Further information about the speed classes of (micro)SD storage cards provides the physical layer specification from the Technical Committee of the SD Card Association. This document is accessible via the URL http://www.sdcard.org/downloads/pls/simplified_specs/archive/part1_301.pdf.

13 Further information about the LAN9512 controller, which contains an USB 2.0 Hub and a 10/100 Ethernet Controller provides the technical specification from the manufacturer. This document is accessible via the URL http://ww1.microchip.com/downloads/en/DeviceDoc/9512.pdf.

14 Further information about the High-Performance Linpack (HPL) benchmark provides the web page http://www.netlib.org/benchmark/hpl/.

15 The Top500 HPL Calculator is accessible via the URL http://hpl-calculator.sourceforge.net.

16 Further information provides the document *HPL Frequently Asked Questions*, which provides the web page http://www.netlib.org/benchmark/hpl/faqs.html.

17 Further information provides the document *Frequently Asked Questions on the Linpack Benchmark and Top500* from Jack Dongarra, which provides the web page http://www.netlib.org/utk/people/JackDongarra/faq-linpack.html.

18 Further information provides the document *HowTo—High Performance Linpack (HPL)* from Mohamad Sindi, which is accessible via the URL http://www.crc.nd.edu/~rich/CRC_Summer_Scholars_2014/HPL-HowTo.pdf.

19 Flops is an acronym for floating-point operations per second.

20 Further information provides the *Technical Reference Manual—Components of the processor—Vector Floating-Point (VFP)* for the ARM1176JZF-S processor, which can be accessed at the URL http://infocenter.arm.com/help/topic/com.arm.doc.ddi0301h/Cegdejjh.html.

21 Further information provides the *Power Measurement Tutorial for the Green500 List*, which can be accessed at the URL http://www.green500.org/sites/default/files/tutorial.pdf.
